# Supplementary material for: Are carbon emissions trading and green financial instruments synergistic? -Comprehensive quantitative research based on content analysis
Source: PLoS One. 2024 Mar 7;19(3):e0298601. doi: 10.1371/journal.pone.0298601 (PMC10919601; doi:10.1371/journal.pone.0298601)
Supplement: S1 Data — (ZIP) [file pone.0298601.s001.zip › Data/地级市Z_F工作报告.zip!/╝¬┴╓/╦╔╘¡╩╨/└┤╫╘╣1⁄2╓┌║┼í╛┬φ┐╦╩2╛▌═°í┐_2012.docx]

各位代表：

　　现在，我代表市人民政府，向大会作政府工作报告，请予审议，并请市政协各位委员和其他列席人员提出意见。

　　一、2011年工作回顾

　　2011年是实施“十二五”规划开局之年，也是松原面对宏观经济形势严峻考验砥砺奋进的一年。在省委、省政府和市委的正确领导下，我们团结和依靠全市各族人民，紧紧围绕统筹推进“三化”、建设幸福松原，逆势攻坚，开拓进取，胜利完成市四届四次人代会确定的主要经济目标，成功扭转金融危机以来全市经济增长放缓的被动局面，实现了“十二五”良好开局。

　　这一年，综合实力迈上新台阶。预计（下同）全市地区生产总值实现1360亿元，净增257亿元，同比（下同）增长15%，是近3年增长最快的一年，经济总量在全省第三的位次上继续壮大。全口径财政收入完成100亿元，净增24.9亿元，增长33.2%，结束了近年来低速徘徊的态势。地方级财政收入完成41.5亿元，净增10.5亿元，增长33.9%，是2007年以来增加额度最大的一年。成功申办第八届全国农民运动会。松原的知名度和影响力进一步提升。

　　这一年，“三化”统筹实现新突破。着眼于做大工业、做美城乡、做强农业，联动推进新型工业、现代农业、现代服务业发展和特色城镇建设。三次产业增加值分别实现225亿元、665亿元和470亿元，分别增长8.5%、15.5%和17.5%。城镇化率提高2.8个百分点。经济社会呈现加快发展的喜人局面。

　　这一年，发展方式转变取得新进展。经济发展加快向投资、消费和出口多元拉动转变，全社会固定资产投资完成803亿元，增长30%；社会消费品零售总额实现385亿元，增长17.5%；实现进出口总额1亿美元，增长13.4%。三次产业比重调整到16.5:48.9:34.6。万元GDP能耗下降3.5%。

　　这一年，人民生活得到新改善。城镇居民人均可支配收入达到18300元，增长8.9%；农民人均纯收入达到7600元，增长23.2%。城乡居民人均储蓄存款余额达到19655元，增长18.3%。城乡居民人均住房面积分别增加到35平方米和25平方米。社会保障提标扩面，人民得到更多实惠。

　　一年来，我们主要做了以下工作：

　　（一）致力于加快发展，全面促进产业升级。全市规模以上工业企业完成增加值520亿元。油气化工产业发展实现突破，松原石油化学工业循环经济园区获准设立，前郭石化资产移交我市。大唐长山热电厂60万千瓦1号机组顺利通过试运行。松原吉安生化实现重组。中国北车集团风电产业园、嘉吉生化3万吨麦芽糊精等114个大项目开工建设。现代农业发展步伐加快。哈达山水利枢纽工程实现库区蓄水，百公里干渠具备通水条件，５台机组并网发电。完成土地开发整理20.9万亩。推广玉米膜下滴灌30.2万亩，新打抗旱井8620眼。粮食产量达到701万吨。前郭县王府站镇现代农业示范区集约化经营模式获得成功。新建标准化规模养殖场（小区）220个，实现畜牧业增加值89亿元。规模以上农产品加工企业达到680户。服务业增速继续保持全省第一。中东新天地购物广场建成运营，金钻、哈萨尔等商圈初具规模。与中青旅合作整体打造查干湖景区进展顺利。全市旅游综合收入实现35亿元，增长28.7%。

　　（二）致力于协调发展，联动推进城乡建设。围绕中心城市建设提档升级，调整完善了城市总体规划，高水平编制了一江两岸、中心城区门户区等专项规划。完成和谐大街新建和中山大街延伸等重点工程。城市建设更加关注百姓所盼，新建城区巷路15条，改造供换热站28座、供热管网120公里，两座大型热源厂二期工程竣工。江南10万吨、江北5万吨两座污水处理厂投入使用。乌兰大街地下人防主体工程封闭。西大嘴子护岸等城区水利工程开工建设。宁江湾游船码头建成运营。高质量完成城区23条街路绿化美化和一廊、三桥、三带等亮化工程。哈大客专扶余段、松陶铁路、绕城高速等重大交通基础设施工程有序推进。县城和小城镇建设步伐加快。建设新农村试点社区50个。新建县乡村级公路430公里，全市行政村晴雨通车率达到98.8%。

　　（三）致力于创新发展，扎实推进改革开放。集体林权制度主体改革结束。全市机构编制核查和中小学教师职称制度改革试点任务圆满完成，成功经验均在全国推广。医药卫生体制改革稳步推进。市政务服务中心新办公楼交付使用，行政审批制度改革取得新进展。城市管理体制改革迈出重大步伐，组建了市容环境卫生管理局。金融创新实现历史性突破，积极开展城镇化金融创新示范试点，20亿元城市基础设施建设融资完成前期审批，新引进４家金融机构，新成立4家畜牧业担保公司。民营经济实现主营业务收入1100亿元，增长24.5%。云天化“六颗星”荣获吉林省公众最信赖商标，“长岭粉条”获国家地理标志证明商标。招商引资成果显著，积极对接中信、中化、中粮、中电投、中视传媒、正大、华电、华润、巴斯夫等大企业，格力电器东北结算中心落户我市，成功引进年产40万吨烯烃等一批重大项目。招商引资到位资金578亿元，利用外资1.4亿美元，分别增长14.9%和12%。

　　（四）致力于全面发展，统筹推进社会事业。开工建设中小学校舍安全工程项目120个，改造薄弱学校30所，市第一高中教学楼、第二高中综合楼等一批教育基础设施工程竣工投入使用。顺利实现平安高考，喜获4个全省单科第一，再度夺得全省理科状元。3个重点科技成果产业化项目获得国家中小企业创新资金支持。中西医结合医院主体工程封闭，3家乡镇卫生院完成改造。市文化“三馆”建成开馆，新建农家书屋、乡镇综合文化站和农村文化大院683个。马头琴制作技艺等3个项目列入国家级非物质文化遗产名录。大型满族新城戏《洪皓》晋京展演成功。《天歌》、《大金始祖》等5部影视作品进入后期制作阶段。完成383个村屯广播电视“村村通”工程。圆满承办“查干湖杯”全国公路自行车冠军赛和全省青少年举重锦标赛。市老年大学教学楼完成改造并投入使用。

　　（五）致力于和谐发展，切实增进民生福祉。全市最低工资标准每月提高170元，企业退休人员基本养老金人均每月提高144元。新增城镇就业6.9万人，安置再就业1.7万人，“零就业家庭”保持动态为零，城镇登记失业率控制在2.9%以内。转移农村剩余劳动力68.4万人。城镇基本医疗保险实现市级统筹，城乡居民医保参保补助标准均提高80元，新农合参合率达到98.5%。免费为全市1008名白内障患者实施复明手术。城镇居民养老保险和新农保试点扩大到三县一区。发放失业保险金1.8亿元，彻底解决下岗失业人员失业保险历史遗留问题。市城区城市居民低保月标准提高50元，市城区农村居民低保年标准提高342元。市场物价稳控在全省最低水平，发放临时价格补贴620万元。发放住房公积金贷款1.7亿元。改造棚户区59.3万平方米，开工建设回迁房116.3万平方米、廉租房15.2万平方米，改造“暖房子”178万平方米、农村泥草房36708户。解决了16.3万农村人口饮水安全问题。实现前郭城区和市区统一供暖，群众满意度大幅提升。信访积案化解成效显著。公安机关清网追逃行动取得重大战果，社会治安状况进一步好转，人民群众安全感不断增强。

　　一年来，民主法制建设深入推进，自觉接受市人大及其常委会的法律监督、工作监督和市政协的民主监督，落实市人大代表议案1件，办理市人大代表建议30件、市政协提案95件，面复率、办复率和满意率均达100%；坚持依法行政，办理行政复议案件43件。全面加强政府自身建设，行政效能明显提升，软环境整治力度加大，双拥共建、审计、统计、档案、质监、食品药品监管、民族、宗教、外事、侨务、气象、地震、残联、地方志等工作都取得了新成绩。

　　各位代表！这一年，面对改革发展稳定的繁重任务，面对全市人民的新期待，我们在挑战中克难奋进，在承继中阔步前行，开创了建设幸福松原的崭新局面！今天的松原，发展思路更加清晰，发展目标更加明确，发展举措更加有力，发展步伐更加稳健，到处涌动加快发展的春潮，上下呈现蒸蒸日上的气象！站在新起点上的松原，充满无限生机和希望！一年来成绩的取得，是省委、省政府和市委统揽全局、正确领导的结果，是市人大和市政协有效监督、大力支持的结果，是全市广大干部群众和社会各界真抓实干、团结拼搏的结果，是离退休老领导、老干部热情关注、积极参与的结果。我代表市人民政府，向所有为松原发展做出贡献的同志们、朋友们，表示崇高的敬意和衷心的感谢！

　　各位代表！在看到成绩的同时，我们也清醒地认识到，当前我市经济社会发展还存在一些比较突出的问题。经济总量不大、产业结构不优、发展质量效益不高的局面还没有从根本上改变；资源开发利用不足，油气化工、农畜产品加工等主导产业支撑拉动作用不强，资源的产地优势还没有转化为产业优势；“三化”统筹进程还需进一步加快，县域经济、开发区经济和民营经济发展潜力尚未充分释放；部分低收入群众生活仍很困难，百姓住房、教育、医疗等方面条件有待进一步改善；社会治安、安全生产等方面还存在薄弱环节，维护稳定任务依然十分繁重；一些部门和公职人员服务意识不强，经济发展软环境亟待优化。对此，我们必须高度重视，采取有力措施，认真加以解决。

　　二、2012年主要工作

　　2012年是我市建市20周年，也是松原继往开来、发展振兴的关键之年。新的起点、新的征程，既充满机遇，也充满挑战。政府工作的总体要求是：按照市五次党代会总体部署，深入贯彻落实科学发展观，坚持以“三化”统筹引领经济社会发展，以建设幸福松原为目标，紧紧围绕加快发展和改善民生，着力做大石化产业、做美一江两岸、做实现代农业、做好特色旅游业，全力维护社会和谐稳定，解放思想，真抓实干，综合提质，持续快上，努力开创松原大发展、新跨越的崭新局面。

　　按照这个总体要求，2012年全市经济社会发展主要预期目标是：地区生产总值实现1605亿元，增长18%；全口径财政收入实现125亿元，地方级财政收入实现50亿元，分别增长25%和20.5%；全社会固定资产投资完成1044亿元，增长30%；社会消费品零售总额完成450亿元，增长17%；城镇居民人均可支配收入达到20800元，农民人均纯收入达到8600元，分别增长13.7%和13.2%；城镇登记失业率控制在4%以内；万元GDP能耗下降3.5%。重点做好以下几方面工作：

　　（一）加快统筹推进“三化”。坚持统筹推进工业化、城镇化和农业现代化不动摇，带动服务业扩量升级，在促进“三化”深度融合中不断提高发展水平。

　　以做大总量为核心，促进工业提速增效。坚持规模化、多元化发展方向，夯实工业发展基础，壮大工业经济实力。新增规模以上工业企业30户。全市规模以上工业实现增加值600亿元，增长15.4%。抓中省直企业扩能。坚持打好“油气牌”，全力支持吉林油田分公司千万吨油气田和东北油气分公司百万吨油气田建设，全市油气产量当量达到780万吨。加快实施长化集团“45-80”扩产改造项目。支持大唐长山热电厂1号机组投入运营，争取启动建设2号机组。抓主导产业壮大。大力发展油气化工产业。全面盘活前郭石化资产，重新启动四氢呋喃装置，加快松原石化200万吨炼油项目建设，启动建设年产40万吨烯烃项目。积极发展农畜产品精深加工产业。推动嘉吉生化10万吨麦芽糊精项目全面达产、启动建设20万吨糖浆项目，促进天原酒精投产运营，争取大成生化100万吨饲料、20万吨蛋氨酸和中粮集团百万头生猪屠宰加工项目建成投产，推动正望油脂、扶余松源玉米深加工等企业实现重组。抓优势产业培育。加快提升装备制造业，开工建设三一工程机械整机与零部件再制造等重点项目，推进北车同力300台风电主机等项目建成投产、奥瑞海山玉米联合收割机等项目全面达效。加快发展新材料产业，开工建设中兴恒和降解塑料等重点项目，推动迦南废旧轮胎资源再生利用等项目建成投产。加快培育新能源产业，开工建设国电龙华风电等项目，推进国能长岭生物质发电等新能源项目投产达效。抓工业效益提升。强化对重点企业调度服务。组织实施“双百”企业技术创新计划和5个重大科技成果产业化项目。大力实施名牌战略，争创7个省级名牌。全市工业利税达到220亿元，税收占市全口径财政收入比重达到80%。

　　以中心城市建设为重点，加快城乡建设步伐。创新城市发展理念，围绕打造具有鲜明特色的中国北方绿色生态休闲宜居城市，加快构建“1139+N”城镇体系，城镇化率达到46%。完善提升城乡规划。按照立足长远、科学引领、市域一体、彰显特色的原则，高标准编制城乡建设规划，不断优化城乡空间布局。重点完善滨江新区规划，全面完成《哈达山旅游经济开发区总体规划》、《天河岛生态景观规划》等各类专项规划，健全完善县（区）、重点镇和新农村规划。深入实施沿江开发战略。着眼于塑造城水相依、人水亲和、生态环境优美、地域文化鲜明的中心城市新形象，以一江两岸为轴线，强力推进滨江新区组团建设、连片开发，着力打造标志性精品工程。加快生态环保型壅水坝建设，进一步改善城区生态环境。突出生态与文化相融理念，精心建设天河岛、纳仁汗、巴尔达、龙华文化等9个公园。高标准建设滨江大道“百米绿廊”，逐步把沿江两岸建成城市“氧吧”。提升城市综合承载力。完成城区南、东两个出入口道路改造。实施滨江大道、前程大路、和谐大街、郭尔罗斯大路等改造延伸工程，启动建设松原大桥与滨江大道连接辅路，完善城市路网。完成松花江大桥维修。做好松花江第三大桥项目前期工作。完成中山广场改造。启动建设江南公交调度中心。完成宁江区客运站建设前期工作。新建改建3个大型热源厂，争取开工建设大唐2×30万千瓦热电联产项目。启动建设江南工业水厂和江南污水处理厂中水工程。加快推进“气化松原”进程。继续推进城区扩容，重点规划建设哈达山新城区、郭尔罗斯新城区和经济技术开发区新区、雅达虹新区，统筹推进江南江北均衡发展，进一步增强中心城市辐射带动力。加快城镇化综合配套改革和城镇服务业发展，促进人口向中心城市集聚。扎实推进中小城镇和新农村建设。加快扶余撤县设市进程，力争年内通过审批。切实抓好乾安、长岭、长山小城市建设。积极打造弓棚子、太平川、查干花等特色小城镇。深入推进新农村建设，加快建设50个新型农村示范社区，推动农村水电路气等基础设施建设和公共事业发展，新建农村公路280公里，促进城镇化与新农村建设良性互动。

　　以特色旅游业为突破口，促进服务业发展提速升级。坚持挖掘潜力、释放能量、培育增量，推动服务业加快振兴。全年实现增加值570亿元，增长21.3%。突出发展特色旅游业。着眼于打造具有重要影响力的中国优秀旅游目的地城市，突出生态和民族特色，推进旅游综合开发。重点打造查干湖、哈达山、泥林、大金碑、龙凤山等旅游景区景点。争取龙华景区晋升为国家4A级景区。逐步开发游江、游岛、观鸟、品三江鱼宴、赏冰雪雾凇等旅游项目，打造沿江生态旅游品牌。谋划开发游原、骑马、野营等特色草原旅游项目，培育新的旅游增长点。加大宣传力度，提升松原旅游影响力，着力办好查干湖蒙古族民俗旅游节、查干湖冰雪渔猎文化旅游节、伯都讷端午文化节、首届旅游文化美食节和生态湖泊论坛等系列活动。全年接待游客400万人次，实现旅游综合收入45亿元。大力提升商贸物流业。加快“三大商圈”建设，完善核心商业区整体功能，哈萨尔购物广场、欧亚购物中心等大型商场投入运营。改造升级三井子、乌兰图嘎等农副产品批发市场。继续落实好国家相关政策，鼓励汽车、文化、休闲、健身等多种消费，积极培育新的消费热点。围绕区位、交通优势，抓好东北亚医药和万隆集散等现代物流园区建设，促进营口港务集团建材物流基地等项目落地，大力发展通道物流经济。支持房地产业健康发展。规范房地产开发市场，完善土地供应机制，优化住房供应结构。完善鼓励住房消费政策，大力激活商品房市场。全市开发房地产375万平方米。加快发展新型服务业态。吸引大型企业集团总部、分支机构或区域研发、管理、销售和结算中心落户松原，争取总部经济发展实现突破。大力发展文化创意、影视制作等经营性文化产业，规划松原文化产业园区和文化商品一条街，争取开工建设中视传媒哈达山冰雪影视基地。加快培育信息服务、会展传媒、电子商务等新兴产业。

　　以农业产业化为导向，大力发展现代农业。巩固农业基础地位，坚持工业化思维和市场化理念，加快传统农业大市向现代农业强市迈进。全年实现增加值245亿元，增长8.9%。提升粮食综合生产能力。完成哈达山水利枢纽工程建设，启动松原灌区建设。加快中低产田改造和盐碱地开发步伐，完成土地整治12万亩，新建高标准基本农田12.5万亩。完成玉米膜下滴灌20万亩。建设全程机械化示范区60万亩。加快推进新一代天气雷达系统建设。粮食产量力争达到800万吨。调整农业产业结构。大力发展特色农业，无公害、绿色、有机农产品种植面积发展到160万亩。加快发展棚菜产业，重点打造宁江区、松原农业高新技术开发区蔬菜生产基地，四县各打造两个百亩蔬菜生产小区，全市棚菜面积突破10万亩。继续抓好6个现代农业产业园区建设。出台扶持畜牧业发展政策，加快建设6个现代畜牧业产业园区，新建标准化规模养殖场（小区）160个。全年实现畜牧业增加值100亿元。大力发展劳务经济，实现劳务收入25亿元。推进农业产业化经营。构建政府、企业、金融机构与农民合作社“四位一体”的现代农业发展模式，加快发展高效农业。推进与吉粮集团、甘肃农垦集团等企业合作，全市土地集约经营面积达到300万亩。新增规模以上农产品加工企业20户、新型农村合作经济组织400个。组建松原粮食产业发展集团，着力培育知名农产品品牌，逐步提高农产品附加值。

　　（二）全力发动三大引擎。强化项目建设促投资、招商引资聚要素、改革开放增活力，形成助推“三化”统筹强劲动力，不断增强发展后劲。

　　以项目建设为支撑，加大投资拉动力度。坚持开发、储备与服务齐头并进，规模、质量与效益整体升级，全力以赴推进大项目建设。着力实施一批重点项目。全市建设投资3000万元以上项目600个。突出抓好工业项目。新开工建设北京利源年产4万吨丁二醇、长春佳辰年产1万吨甲基呋喃等重点工业项目100个，其中1-3亿元项目20个、3-5亿元项目8个、5－10亿元项目6个、10亿元以上项目2个。加快实施交通基础设施项目。扎实推进松陶铁路建设。抓好绕城高速续建工程，国道203线拐脖店至长岭段二级路建成通车，加快推进省道304线前七号至科左中旗段二级路等改建项目，完成长乌铁路长岭段、五右高速松原段建设和国道102线乌金屯至北沟二级路改造前期工作。争取松原内陆港开工建设、松原机场完成规划审批和立项计划。科学谋划一批重大项目。设立项目谋划专项资金。根据国家产业走向和政策导向，坚持产业项目和基础设施项目、民生社会事业项目并重，谋划一批高科技、高附加值、高回报率、高就业率大项目。全市新储备各类亿元以上项目200个，其中产业类项目达到160个。强化项目服务管理。深入落实重大项目包保责任制，建立重大项目“绿色通道”，健全完善项目建设考核机制，切实提高项目开工率、建成率、投产率和达效率。

　　以招商引资为主线，努力破解资金瓶颈。树立大招商理念，设立招商引资专项资金，营造浓厚招商氛围，坚决打好招商引资攻坚战。实现招商引资到位资金700亿元，增长21%。全方位增强招商引资实效性。立足资源优势，突出主攻方向，重点围绕培育和延伸油气化工、农畜产品加工、生物化工、机械制造等产业链条，着力与百胜、杜邦、盼盼、三一重工等大企业联系对接，有针对性地开展产业招商和专项招商，在引进世界500强、中国500强和中国行业50强等战略投资者上实现重大突破。争取格力集团新元电子电容器、来禾化学70万吨总溶剂等一批大项目落地。多渠道畅通金融血脉。深化与开发行、农发行、深圳正大生命人寿等金融企业合作，着力抓好城市基础设施建设融资项目推进落实。促进市国有资产经营管理公司与吉林九台农商行合作，实现国有资产保值增值。加大土地收储融资力度。积极引进光大、民生等金融机构。进一步发挥市担保公司平台作用，争取新增小额贷款公司和各类担保公司12家。力争5户企业纳入全省上市培育工程。深入推进城镇化金融创新示范试点工作，积极探索现代融资新模式。优化金融生态环境，促进银企有效对接。

　　以改革开放为动力，增强经济发展活力。认真总结建市20年发展经验，坚持以改革的思维、开放的理念，寻求松原发展新突破。加快推进体制机制改革。扎实做好分类推进事业单位改革基础工作。深化财政体制改革，推行部门预算、国库集中支付和预算绩效管理。推进医药卫生体制改革，认真抓好公立医院改革试点。积极稳妥地实施文化广电新闻出版行政体制和国有文艺院团体制改革，组建广播电视台。积极推进户籍管理制度改革。鼓励有条件的乡村进行土地股份制改革。加快集体林权配套改革进程。实施水务管理体制改革，实现水务管理一体化。深入推进行政审批权相对集中改革，进一步提高审批效率。全面提升对外开放水平。争取我市列入全国老工业基地调整改造和全国资源型城市可持续发展重点规划城市，积极寻求与省内其他中部城市错位发展、融合互动。抓住成功申办全国农民运动会有利契机，充分利用东博会、农博会和影视摄制等有效载体，全方位开展对外宣传推介，不断提升松原知名度和影响力。大力发展外向型经济，实现进出口总额1.15亿美元，增长15%。

　　（三）强力扩张三大经济板块。充分挖掘县域经济、开发区经济和民营经济三大板块发展潜力，着力培育支撑“三化”统筹、促进总量扩张的三个重要经济增长极。

　　以争先晋位为目标，加快做大县域经济板块。把“县域突破”置于更加突出的战略位置，放大县域发展优势，促进县域发展提速。加快统筹推进县域“三化”，立足各县资源禀赋和产业基础，集中力量建设各具特色的工业集中区和农畜产品生产加工基地，形成各自发展优势和整体组合优势。争取前郭县纳入省县域“三化”统筹试点，扶余、长岭、乾安在全省县域“三化”统筹中升级晋位。突出抓好大项目建设，各县分别建设亿元以上工业项目5个，其中5亿元以上工业项目至少1个。制定支持县域发展政策措施，在项目建设、招商引资等方面加大支持服务力度，鼓励以存量资产注入和国有土地置换等方式做大各县融资平台。县域地区生产总值实现1290亿元，力争占全市经济比重突破80%。

　　以打造发展高地为方向，加快做优开发区经济板块。制定支持开发区发展政策，加快实现由分散建项目向集群做产业转变。完成松原石油化学工业循环经济园区和哈达山旅游经济开发区组建。加快松原经济技术开发区晋升为国家级开发区进程，促进玉米深加工和机械制造等产业集聚，规划建设食品工业园。加快松原农业高新技术开发区申报国家农业（蔬菜）科技园区和市农科院筹建步伐，推进现代农业高新技术集成基地建设。推动查干湖旅游经济开发区与中青旅深度合作，着力培育环查干湖经济带，争取国家5A级旅游景区和国家生态旅游示范区创建取得实质性进展。探索公司化经营模式，建立高效融资平台，增强开发区自我“造血”功能。开发区经济总量突破225亿元，占全市经济比重达到14%。

　　以政策扶持为依托，加快做强民营经济板块。深入实施新一轮民营经济腾飞计划，全面落实中央支持小型微型企业发展金融财税政策，最大限度扩张民营经济总量。出台扶持中小企业加快发展政策措施，对民营企业在配套投资、贴息补助、融资担保、资金奖励上给予更多支持。着力发展产业型、创业型、科技型孵化基地。继续开展“万民创业小额担保贷款”活动，积极破解中小企业融资难问题。全年实现主营业务收入1380亿元；实现增加值770亿元，占全市经济比重达到47%。

　　（四）努力建设幸福松原。顺应全市人民对幸福生活的新期待，突出解决群众最关心、最直接、最现实的利益问题，努力建设松原人民的幸福家园。重点做好十方面民生实事。

　　一是积极扩大城乡就业。继续开展全民创业带动就业活动，加强公共就业服务，开发公益性岗位899个，力争发放小额担保贷款8500万元。新增城镇就业5.3万人，保持城镇“零就业家庭”动态为零。转移农村剩余劳动力70万人。二是提高社会保障水平。将城镇居民养老保险和新农保试点范围扩大到所有县（区），提前实现养老保险制度城乡全覆盖。继续扩大城镇基本医疗、工伤、失业及生育保险覆盖面。积极做好“五七家属工”后续参保工作。新农合参保补助标准提高40元。三是加大社会救助力度。规范低保管理，进一步提高城乡低保标准和补助水平。积极开展助老、助医、助困、助学和助残等救助项目，继续组织实施“健康快车”复明和“互助关爱助残”等工程，市残疾人康复中心和托养中心一期工程建成使用。筹建市社会精神病院门诊楼。四是大力实施安居工程。建设公租房16.2万平方米、廉租房17.5万平方米，改造城市棚户区51.5万平方米，发放住房补贴2905万元。加大农村危旧房改造力度。改造“暖房子”240万平方米，撤并改造小锅炉房30座，改造供热管网139公里，新增集中供热能力400万平方米。五是着力改善人居生态环境。高标准绿化美化松原大地，完成中山大街等8条道路和苏力德湿地等9个公园绿化，实现松原大路、乌兰大街等20条街路绿化升级。积极创建国家森林城。全面完成主要污染物减排任务。认真执行松花江休养生息政策。建设万隆再生资源回收基地。实施城市精细化管理，加强农村环境综合整治，努力增强人民群众的舒适感。六是认真抓好食品供应保障。提高价格调节基金使用效益，建立市级猪肉储备制度，扩大本地反季节蔬菜市场占有份额，完成松江蔬菜水果批发市场改建和西郊蔬菜批发市场、倪窑农贸大市场改造升级。实施蔬菜批发补贴政策，积极稳控市场物价，保障城乡居民特别是低收入群体基本生活不受影响。加强食品流通链条管理，完善监测评估、检验检测体系，提高食品优质率。加大食品及药品安全专项整治力度，努力保障全市人民吃得放心、用得安全。七是推进公共服务水平升级。进一步提升人口和计生工作水平，提高出生人口素质，促进人口均衡发展。新建改建城乡社区服务中心38个。争取我市列入全国家政服务体系试点城市。优先发展公共交通，开通江南公交调度中心至江北车管所公交线路，完成100座城区新型高档公交站亭建设，市区内伤残军人和70岁以上老人实现免费乘坐公交车。建立健全市场准入和退出机制，规范出租汽车行业管理。市规划展览馆免费向市民开放。规划筹建市青少年宫。完成郭尔罗斯大路人防续建工程。开工建设地震前兆动植物观测站。新建垃圾中转站20座、公厕20个。实施10个城农电网新建扩建项目。解决5万农村人口饮水安全问题。八是推进教育全面均衡发展。加快发展学前教育、职业教育和高等教育。市幼教中心和市特殊教育学校竣工投入使用，力争全面完成中小学校舍安全工程，建成市级示范性初中及小学20所、优秀村小20所，改造薄弱学校25所。启动实施农村小学科学实验室配备项目。九是强化医疗卫生服务。启动市中心医院异地重建，开工建设市生殖保健医院和3个社区卫生服务中心，力争市中西医结合医院投入使用。继续加强重大疫病防控。十是加快发展文化体育事业。积极扶持公益性文化事业发展，鼓励开展广场和社区文化活动，抓好首批国家公共文化服务体系农民自办文化示范项目创建，办好首届农民文化艺术节。规划筹建市文化活动中心和市多功能数字影院。建设农村文化大院183个。送戏下乡480场，放映电影1260场。加大广播电视“村村通”工程实施力度。广泛开展全民健身活动。加强社区和乡村体育设施建设，市体育馆主体竣工，完成市体育场、综合比赛训练馆、游泳馆和短道速滑馆规划立项。积极做好全国农民运动会筹办工作。

　　（五）加强和创新社会管理。积极适应社会结构、社会环境新变化，不断提高社会管理科学化水平，确保社会既充满活力又和谐稳定。

　　完善基层群众自治制度，深入开展政务、村务、厂务公开。着力从政策和制度层面统筹解决信访问题，完善机制，加大投入，切实解决群众反映强烈的热点难点问题。扎实开展“六五”普法，不断完善法律援助和人民调解体系。依法管理宗教事务，积极增进民族团结。加强流动人口和特殊人群的管理服务，提高动态管理水平。全面加强社会治安综合治理，严厉打击各种犯罪活动。进一步提高办案水平，命案破案率达到95%以上，刑事案件破案率、治安案件查结率等均大幅提升。加强校园周边环境和校车安全整治，营造平安和谐的育人环境。牢固树立安全发展理念，狠抓重点行业和领域安全隐患排查整治，坚决保护人民生命财产安全。加强网络信息监管，提高对虚拟社会的管理水平。强化应急管理，加强应急演练，进一步提高突发事件预测预警和应急处置能力，最大限度减少各类灾害、事故造成的损失。

　　三、努力建设服务型政府

　　牢固树立为民执政理念，主动适应新形势、新任务需要，不断加强政府自身建设，努力建设人民满意的服务型政府。

　　（一）坚持依法行政，增强政府公信力。严格依照法定权限和程序行使权力，进一步规范行政行为。坚决执行市政府重大事项议事规则，坚持集体决策、专家论证和社会公示等制度。主动接受市人大及其常委会的法律监督、工作监督和市政协的民主监督，认真执行人大决议决定，坚持重大事项向人大报告、向政协通报制度，广泛听取人大代表、政协委员和民主党派、工商联、无党派人士、社会各界人士的意见和建议。坚持面复制度，认真办理人大代表建议和政协提案。加强政务诚信建设，全面提升政府公信力。

　　（二）坚持为民执政，增强政府感召力。深入基层问政于民、问需于民、问计于民，真诚倾听群众呼声，真情关心群众疾苦，真心解决群众困难。加强政府网站建设，完善新闻发布制度，保障公民的知情权、参与权和表达权。强化市、县（区）长信箱和公开电话职能建设，认真办理群众来信来访，切实解决人民群众合理诉求。推进公共资源向民生领域倾斜、社会保障向弱势群体倾斜，让困难群众得到更多帮助。

　　（三）坚持高效理政，增强政府执行力。始终保持奋发有为的高昂斗志，勇于担当，激情干事，努力争创一流业绩。狠抓作风转变和工作落实，真正把精力放在干事业上，把本领用在促发展上。敢想敢干、大胆突破，鼓励创新、宽容失败，切实增强谋划发展、统筹发展、推动发展、优化发展的本领。坚持简政放权，优化工作流程，完善绩效管理，加大治庸、治懒、治散力度，进一步提高政府执行力。

　　（四）坚持从严治政，增强政府约束力。认真落实党风廉政建设责任制，切实履行“一岗双责”，建立健全惩治和预防腐败体系。强化行政监察和效能监察，健全工程招投标、资源出让、政府采购等重大事项监督约束机制，着力从源头上预防和治理腐败。认真开展政行风和软环境民主评议，严肃查处各类涉软案件，坚决纠正损害群众利益不正之风，加大行政问责力度，努力营造风清气正的政务环境。

　　各位代表！年轻的松原正处在跨越发展的关键节点。统筹推进“三化”、建设幸福松原，是全市人民的深情重托，是历史赋予的神圣使命。新任务光荣艰巨，新形势催人奋进。让我们在市委的坚强领导下，解放思想，昂扬奋进，扎实苦干，开拓创新，以优异的成绩向建市20周年献礼！
